# Effect of omega-3 fatty acids on TH1/TH2 polarization in individuals with high exposure to particulate matter ≤ 2.5 μm (PM2.5): a randomized, double-blind, placebo-controlled clinical study

**DOI:** 10.1186/s13063-022-06091-5

**Published:** 2022-02-24

**Authors:** Xiaomin Wang, Shuiqin Li, Yongcan Wu, Demei Huang, Caixia Pei, Yilan Wang, Shihua Shi, Fei Wang, Zhenxing Wang

**Affiliations:** Hospital of Chengdu University of Traditional Chinese Medicine, No. 39 Shi-er-qiao Road, Chengdu, 610072 Sichuan Province People’s Republic of China

**Keywords:** Ambient particulate matter, Omega-3 fatty acid, TH1/TH2 polarization, Immune system, Randomized controlled trial

## Abstract

**Background:**

Long-term exposure to high concentrations of PM2.5 may cause immune system dysfunction and damage to the respiratory and cardiovascular systems. PM2.5 may cause CD4 + T helper cells to polarize toward TH1 or TH2 cell types, which may be associated with the onset and progression of many human diseases. Recent studies have shown that omega-3 fatty acids can regulate human immune function and reduce physiological damage caused by air pollution; however, only limited research has examined the therapeutic effects of omega-3 fatty acids on subjects with high exposure to PM2.5 in mass transit systems such as subways.

**Methods:**

This study was designed as a prospective, randomized, double-blinded (to participants and researchers), placebo-controlled clinical trial. The research plan is to randomly select 120 eligible adults based on the difference in PM2.5 exposure in the Chengdu subway station. They should be aged 20–65 years old and work in the subway station more than or equal to 3 times a week, each time greater than or equal to 8 h, and had worked continuously in the subway station for more than 2 years. All participants will receive omega-3 fatty acids or placebo for 8 weeks. The primary outcomes will be changes in the TH1/TH2 cell polarization index and changes in serum cytokine concentrations. Secondary outcomes will be changes in early indicators of atherosclerosis, pulmonary function, COOP/WONCA charts, and scores on the Short-Form 36 Health Survey for quality of life. Results will be analyzed to evaluate differences in clinical efficacy between the two groups. A 6-month follow-up period will be used to assess the long-term value of omega-3 fatty acids for respiratory and cardiovascular disease endpoints.

**Discussion:**

We will explore the characteristics of the TH1/TH2 cell polarization index in a population with high exposure to PM2.5. Omega-3 fatty acids and placebo will be compared in many ways to test the effect on people exposed to PM2.5 subway stations. This study is expected to provide reliable evidence to support the promotion of omega-3 fatty acids in clinical practice to protect individuals who are highly exposed to PM2.5.

**Trial registration:**

Chinese Clinical Trial Registry ChiCTR2000038065. Registered on September 9, 2020

**Supplementary Information:**

The online version contains supplementary material available at 10.1186/s13063-022-06091-5.

## Background

With the rapid development of urbanization and industrialization in many countries, air pollution has become an important factor that negatively affects the health of residents in these countries [1–4]. In fact, air pollution has been identified as the cause of death for ~ 2 million people worldwide [5]. Particulate matter (PM), one of the main components of polluted air, is chemically complex and may contain biological compounds, organic compounds, and metals [5]. Studies have found that the composition of PM changes with time and space [6]. PM2.5 is a class of fine particulate matter with a diameter of < 2.5 microns. Unlike coarse PM, fine PM may reach the terminal bronchioles and alveoli of the lungs, causing oxidative stress, oxidative damage, and inflammatory reactions in epithelial cells, as well as persistent adverse effects to the human body [7–10].

The subway is a public transportation system widely used in many major cities throughout the world. As a relatively enclosed space, the PM2.5 concentration inside the subway system is several times higher than that on the street surface above [11]. PM2.5 in the subway is generated by friction between the wheels and rails, wear on the electric rails and overhead lines, electric current collectors, and arcs. PM2.5 is rich in metal elements, including iron, manganese, chromium (from rails/wheels), barium (from brakes), and copper (from electrical components) [12, 13]. Any PM2.5 that settles on the subway floor will become re-suspended in the air due to train and passenger movements, which result in a continuous circulation of PM2.5 within the subway system. Subway workers often experience long-term occupational exposure to high concentrations of PM2.5, which may lead to adverse health effects.

Located in southwestern China, Chengdu is a modern metropolis with a total population of more than 16 million. The Chengdu Metro, which has been in operation for more than 10 years, currently has 8 lines, with a total length of 358.235 km and 215 stations. In Chengdu, more than 1.4 billion commuters ride the subway each year (the Chengdu Metro Line Map is shown in Additional file 1). Previous studies have shown that short-term exposure to PM2.5 in subway systems may cause harm to humans [14]; however, few clinical studies have examined the effects of long-term exposure to PM2.5 among subway workers. To our knowledge, previous studies have mainly focused on physiological damage to humans caused by outdoor air pollution. Studies on subway employees exposed to PM2.5 are very rare. One study in China showed that the concentration of PM2.5 in the Chengdu subway was significantly higher than PM2.5 concentrations detected on city streets [15]. As the number of employees in the Chengdu subway system continues to increase, research is needed to assess potential harms that may result from PM2.5 exposure.

Omega-3 polyunsaturated fatty acids (PUFA), including eicosapentaenoic acid (EPA), docosapentaenoic acid (DPA), and docosahexaenoic acid (DHA), have been shown to produce a variety of beneficial effects on human health [16]. A large number of epidemiological studies and clinical trials have shown that consumption of omega-3 fatty acids may have beneficial effects by reducing inflammation and regulating the function of macrophages, neutrophils, T cells, and B cells [17, 18]. Studies have found that omega-3 fatty acids may regulate immune function and have beneficial health effects in clinical trials examining PM2.5 in indoor air pollution [19–21].

PM2.5 may damage the human body through systemic inflammation, changing the immune response, and increasing oxidative stress [22]. As the initial deposition site of PM2.5, airway damage includes inflammation, bronchial remodeling, and tissue fibrosis, which often appear in the early stages of disease [23]. PM2.5 exposure may disrupt the balance of TH1 and TH2 helper cells, cause the TH1/TH2 balance to shift in the direction of TH2 cells, and increase the incidence of bronchial asthma and chronic inflammatory diseases [24, 25]. Although PM2.5 exposure is known to adversely affect TH1/TH2 polarization in humans, few clinical trials evaluating the impact of omega-3 fatty acids on TH1/TH2 polarization during PM2.5 exposure have been conducted. Therefore, this study was designed as a prospective, randomized, double-blinded (to participants and researchers), placebo-controlled trial to objectively evaluate the effects of omega-3 fatty acids on TH1/TH2 polarization in subway workers exposed to PM2.5.

## Methods

### Study design

**Fig. 1:**
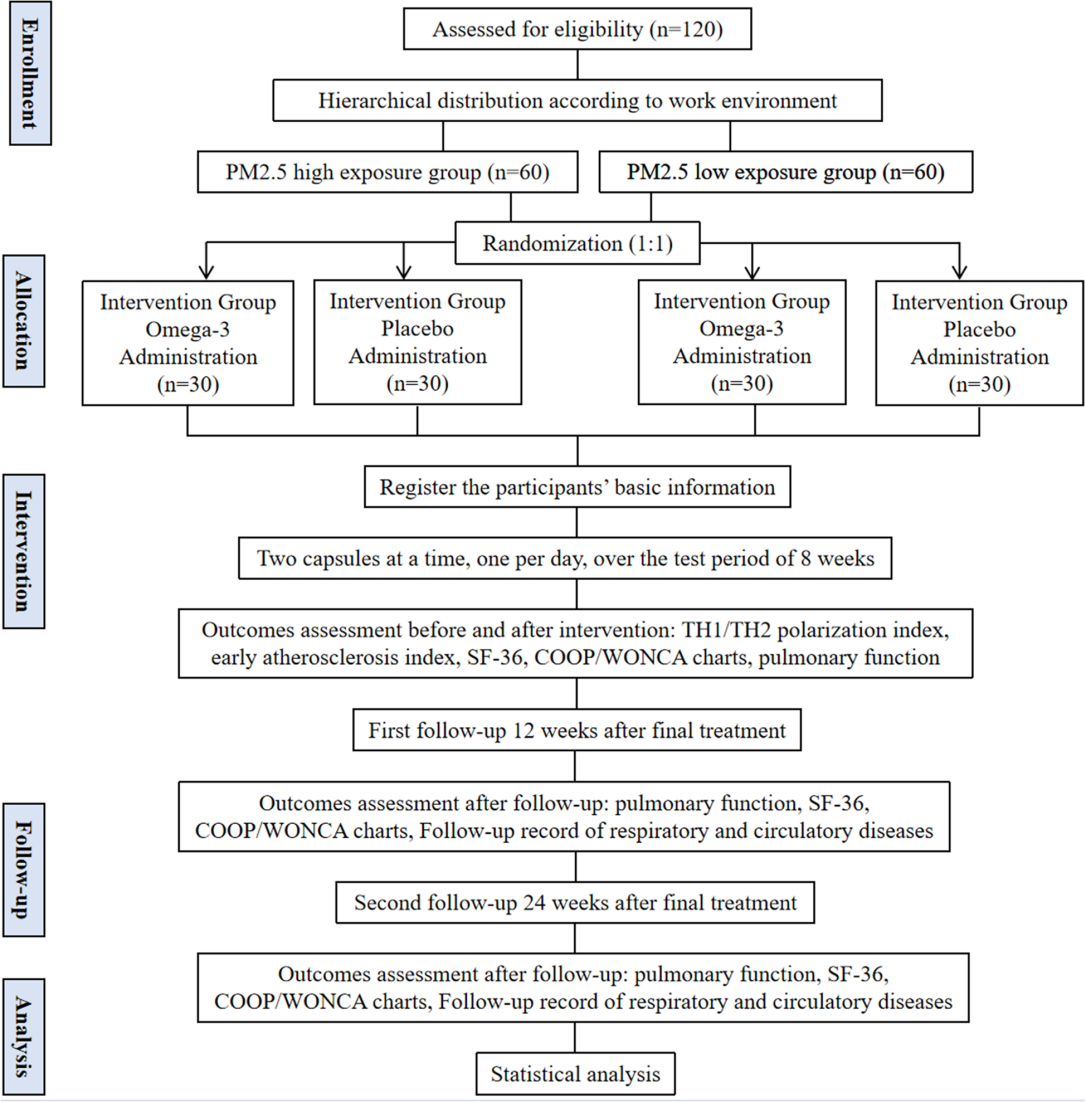
Study flowchart

This is a randomized, single-center, placebo-controlled, double-blinded clinical trial. This research protocol complies with the standard protocol project: Interventional Trial Recommendations (SPIRIT) Guidelines (Additional Document 2). Eligible participants will be randomized 1:1 into two groups: the intervention group and the placebo group. Random numbers were generated by IBM SPSS Statistics V.25 software. The TH1/TH2 cell polarization index in the serum will be collected to determine the potential impact of people with high PM2.5 exposure. The Hospital of Chengdu University of Traditional Chinese Medicine (Sichuan Province, China) will serve as a research institution to the Chengdu Metro Company to recruit participants. The flowchart of this trial procedure is shown in Fig. 1. This study protocol has been approved by the Chinese Ethics Committee of Registering Clinical Trials (Ethical Review No.: ChiECRCT20190343). Before randomization, all eligible participants will be asked to sign an informed consent (see Additional file 3).

### Participant recruitment

We plan to recruit 120 participants from the Chengdu Metro Station. The recruitment methods used in this study include (1) face-to-face communication, (2) bulletin boards, and (3) posters. We will post recruitment information at various subway stations in Chengdu and broadcast the recruitment information on the subway broadcast. The members of the research team will receive uniform training after the project is launched, all of whom are medical staff with doctor certificates. The members of the research team will obtain the written consent of the participants, and their mobile phones will be kept open for 24 h to communicate with the participants at any time. Participants can enter clinical trials only after the members of the research team confirm the qualifications of the participants on the spot and sign a written informed consent form. Eligible participants will be registered and randomly assigned to placebo or omega-3 fatty acid treatment. There is no anticipated harm and compensation for trial participation. All subject personal information and trial data will be treated as confidential, which will be recorded in the individual case report forms (CRFs). These data will be uploaded in a database that only members of the research team can access. After the later data was completely uploaded, the subjects can enter the website (http://www.medresman.org.cn/login.aspx) to check. Enrollment will begin on November 1, 2020, and is expected to be completed by July 31, 2021.

### Sample size

G*Power 3.1 will be used to calculate the sample size necessary to have sufficient statistical power [26]. The sample size calculation will be based on a previous study of omega-3 fatty acids and their effects on up-regulating the TH1/TH2 ratio by ~ 32.5% [27]. The type I error is 0.05 and the power is 80%. Considering a dropout rate of 20%, we aim to recruit 120 subjects. The treatment group and the placebo group were assigned 60 participants each. Based on the grouping of our experiment, we will be divided into the high exposure group and low exposure group according to PM2.5 exposure. In the high and low exposure groups, 30 subjects were treated with omega-3 fatty acids and placebo.

### Selection criteria

Participants for this study will be recruited from long-term employees of the Chengdu subway system, who do not routinely wear professional anti-PM2.5 masks.

#### Inclusion criteria for the high PM2.5 exposure group

1. Employees of the Chengdu subway system, regardless of gender, 20–65 years of age, who work in one of the station halls ≥3 times per week, ≥8 h per day, and have been on the job continuously for ≥2 years
2. No obvious abnormalities detected by a physical examination in the past 1 year
3. Have not received hormones, antioxidant supplements (vitamin C, vitamin E), or non-steroidal anti-inflammatory drugs in the past 3 months
4. No history of surgery in the past 3 months
5. Willing and mentally competent to consent to participate in this experimental study and to sign an informed consent document

#### Inclusion criteria for the low PM2.5 exposure group

1. Employees of the Chengdu subway system who work in offices (with an air-conditioning device equipped with PM2.5 filters), regardless of gender, 20–65 years of age, who work in the office ≥3 times per week, ≥8 h per day, and have been on the job continuously for ≥2 years
2. No obvious abnormalities detected by a physical examination in the past 1 year
3. Have not received hormones, antioxidant supplements (vitamin C, vitamin E), or non-steroidal anti-inflammatory drugs in the past 3 months
4. No history of surgery in the past 3 months
5. Willing and mentally competent to consent to participate in this experimental study and to sign an informed consent document

#### Exclusion criteria

1. A long-term history of drug use
2. Routine use of a PM2.5 respirator that complies with China’s “PM2.5 Protective Masks” group standard (TAJ 1001-2015) during work
3. A history of smoking
4. A history of bleeding
5. An allergic reaction to flaxseed, fish, and/or seafood
6. A history of hepatitis, fatty liver, cirrhosis, cholecystitis, or gallstones
7. Currently pregnant or lactating
8. Participation in another clinical trial within the past 6 months
9. Inability to understand the intention of the experiment and inability to cooperate with the experimental procedure

### Randomization, allocation, and concealment

Before the subjects agree to participate in this study, all qualified subjects who satisfy the inclusion and exclusion criteria will be given an information sheet describing the study and an opportunity to ask questions and clarify their concerns with the investigators. During the study, both the participants and the researchers will be blinded to the grouping information for each subject based on the PM2.5 exposure level. According to the difference in PM2.5 exposure, 120 random numbers will be generated by BMISPSSStatistics24.0 software, and 60 random numbers will be allocated to the PM2.5 high-exposure group and PM2.5 low-exposure group (stratified random). The researcher in charge of blinding will use two opaque envelopes to seal the random numbers and will carefully check whether the serial numbers and drug numbers on the cover and letterhead are consistent with the labels on the drug packaging. Each subject will be provided with a corresponding emergency envelope. In case of an event threatening the safety of the subject, the researcher will allow the unblinding in an emergency after the evaluation so that the patient can be properly treated. In an emergency, an independent pharmacist will open the sealed envelope and notify the investigator of the assignment. Information about the date, time, and reason to remove blindness will be entered in clinical case forms (CRFs) and envelopes.

### Intervention

We plan to recruit 120 subjects who will be automatically divided into two groups based on their PM2.5 exposure level in the work environment: high PM2.5 exposure group (*n* = 60) and low PM2.5 exposure group (*n* = 60). In the high-exposure group, 30 people will receive omega-3 fatty acid treatment and the other 30 subjects will receive placebo treatment. Participants in the low-exposure group will receive an identical treatment regimen.

1. Intervention group: Participants will receive treatment from the same batch of omega-3 fatty acids (1200 mg/capsule) provided by Kirkland, Canada. Participants will take two capsules every morning for 8 weeks and will be followed for 24 weeks after the trial. The omega-3 fatty acid capsules will be stored in a dry environment at room temperature.
2. Placebo group: Participants will receive placebo treatment, containing soybean oil as the main ingredient, which has been produced by the Placebo Experimental Center of the School of Pharmacy, Chengdu University of Traditional Chinese Medicine. The physical appearance and weight of the placebo will be the same as the omega-3 fatty acid capsules, and the administration methods, experimental period, and storage methods will be identical to those in the intervention group.
3. The capsules for the intervention group and the placebo group will be packaged in the same box. Each box will contain 8 weeks of drug dosage and will include the name of the subject, dosage, consumption schedule, description of storage conditions, and expiration date. “Experiment only” will be highlighted in a prominent position on the box. We have selected soybean oil as the placebo (comparison) agent because it is commonly used in daily cooking, its physical properties are similar to omega-3 fatty acids and it has no obvious effects on TH1/TH2 polarization [28].
4. The drug to be used in this project is a nutritional supplement with controllable safety risks. The participant may do so if he/she has no adverse events or side effects. However, if the subject has diarrhea or nausea, their participation in the trial may be suspended and the adverse event will be reported to the research team. Due to the special nature of omega-3 fatty acids, we will prohibit participants from using antioxidant supplements, such as vitamin E, during the trial. Medications used by participants to treat other diseases will not be curtailed or changed. If omega-3 fatty acids are beneficial to subjects with high PM2.5 exposure, we will put the control subjects on the “waiting list,” that is, after the trial, the control subjects will also be provided with omega-3 fatty acids for 8 weeks free of charge.

### Data and sample collection

We will use the Chinese Clinical Research Public Management Platform (Res Man) to collect and manage the data. The experimental data can only be accessed and manipulated by the research team.

The person in charge of the project will have access to the real-time data, but cannot make any changes to the data.

**Fig. 2:**
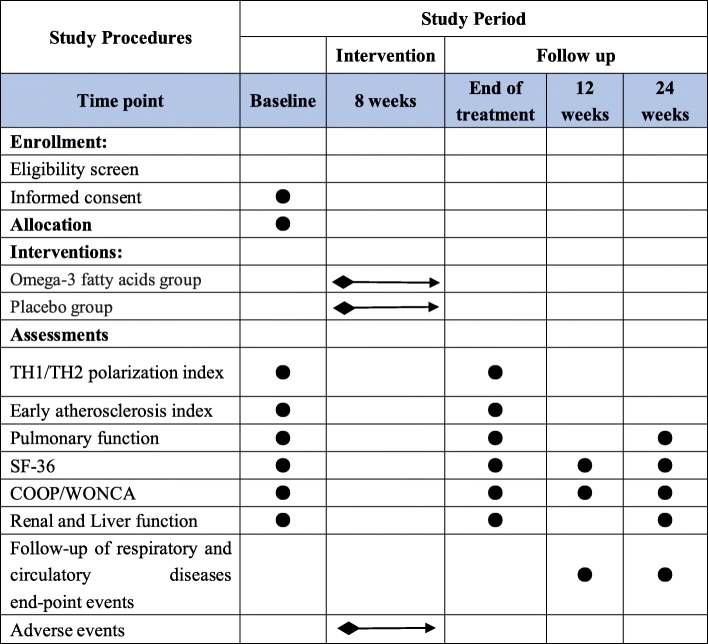
Participant timeline

All data collectors will receive uniform training to ensure that data collected through questionnaires will have high quality and consistency. The Short-Form Health Survey 36 (SF-36) [29], designed to assess the quality of life through a comprehensive assessment of physical, mental, and social components and COOP/WONCA charts, will be completed at baseline, immediately after the final treatment, and then at 12 weeks and 24 weeks after the final treatment. A pulmonologist will collect data on pulmonary function from all subjects to ensure consistency prior to and following the final treatment, as well as 24 weeks after the final treatment. We also will examine the follow-up records for each patient regarding respiratory and cardiovascular diseases at 12 weeks and 24 weeks following treatment. The time frame for data collection and assessments is shown in Fig. 2.

The number of TH1 and TH2 cells in the peripheral blood of subjects and the TH1/TH2 ratio will be measured at the time of enrollment (baseline) and again following treatment (8 weeks later). Similarly, we will determine the TH1/TH2 polarization state, including the concentrations of serum interferon-γ (IFN-γ), interleukin-4 (IL-4), interleukin-12 (IL-12), interleukin-6 (IL-6), interleukin-8 (IL-8), and intercellular adhesion molecule-1 (ICAM-1), as well as liver and renal function to assess drug safety, at baseline and following treatment.

The concentrations of TH1 and TH2 cells in the peripheral blood immune cell population will be detected by flow cytometry using a CytoFLEX Flow Cytometer (Beckman Coulter, Brea, CA, USA). Serum IFN-γ, IL-4, IL-12, IL-6, IL-8, and ICAM-1 levels will be measured using commercially available enzyme-linked immunosorbent assay (ELISA) kits. The nurse will collect the venous blood of the subjects, some will be stored in a 4 °C ice pack and immediately sent to the laboratory for testing (liver function and renal function), some will be stored at room temperature and sent to the laboratory for flow cytometry testing, and a part was frozen at −80 °C for ELISA detection. All blood samples will be assessed in the Laboratory of Hospital of Chengdu University of Traditional Chinese Medicine. The blood products of all subjects will be destroyed uniformly after the end of the study.

### Outcome measures

#### Primary outcome measure

At enrollment and 8 weeks later, we will evaluate the concentrations of cells about TH1 and TH2 in peripheral blood and the TH1/TH2 polarization state (IFN-γ, IL-4, IL-12, and IL-6) and calculate the TH1/TH2 ratio using the IFN-γ/IL-4 balance index. We will examine the difference in the concentration of TH1 and TH2 cells in the peripheral blood and the TH1/TH2 polarization state between the treatment group and the placebo group.

#### Secondary outcome measures

We will evaluate the difference in secondary indicators between the treatment group and the placebo group from the following four aspects.

1. A pulmonary function test will be performed to evaluate the impact of PM2.5 on lung function in each subject (baseline, end of treatment, and 24 weeks).
2. Early indicators of atherosclerosis, including IL-8 and ICAM-1, will be measured (baseline and end of treatment).
3. The SF-36 questionnaire will be used to assess the health status of each subject and to generate a COOP/WONCA chart. The quality of life will be summarized for eight aspects: physical functioning, physical role functioning, physical pain, general health, vitality, social functioning, emotional health, and mental health (baseline, end of treatment, 12 weeks, and 24 weeks).
4. Safety and adverse reactions to drug treatment will be monitored.

### Adverse events’ reporting and safety monitoring

The omega-3 fatty acids used in the project are a nutritional supplement with controllable safety risks. The Chinese Ethics Committee of Registering Clinical Trials (Ethical Review No.: ChiECRCT20190343) has approved this study. If the subject has any adverse events related to this study (such as diarrhea or nausea), it will be reported to the research team and recorded in the CRF. The project supervisor will also collect adverse events in a timely manner and decide on follow-up treatment (including close observation, additional medical management, or early termination).

### Data management and monitoring

Data collection and monitoring will be managed by a dedicated Data and Security Monitoring Board (DSMB). The DSMB will be composed of a deputy chief physician from the respiratory department, a junior Chinese medicine practitioner, and a statistician, and these people are independent from the sponsor and competing interests. DSMB members will provide appropriate recommendations on the safety and completeness of all procedures during the clinical trial. They will occasionally monitor the progress of the research and ensure that the rights and well-being of the subjects are safeguarded. All completed questionnaires and laboratory results will be stored in a locked cabinet. The data will only be accessible to the researchers. Two data entry clerks will enter all data into an electronic database at the same time. The electronic database will be maintained as a password-protected file.

### Adherence to study interventions

Researchers will use varieties of strategies to encourage and monitor compliance with research interventions. During the 8-month research observation period, we will provide clear written instructions for the participants. The content will consist of adherence and lifestyle assessment guidelines to daily research capsule intake. If necessary, we will weekly contact all participants to evaluate how they manage the intervention and provide further personalized guidance. An independent drug administrator will take care of dispensing, recalling, storing, and recording all tested drugs.

### Statistical methods

IBM SPSS Statistics V.25 software will be used for statistical analysis of the data collected in this study. Subjects who withdrew from the experiment but received at least one treatment will still be included in the intention-to-treat analysis. We will use various attribution methods to conduct sensitivity analysis to test whether the results are reliable for different assumptions about missing data. Demographic and baseline data will be tabulated and evaluated using analysis of variance (ANOVA) or the *χ*^2^ test. Analysis of variance was performed on categorical variables, and the Pearson *χ*^2^ test was performed on continuous variables. Subgroup analyses will be performed according to the subjects’ working years (2–6 years, 6–10 years, and over 10 years) and average working hours per week (24–36 h and over 36 h). If conditions are appropriate, subgroup analysis will be performed with reference to the above criteria. 95% CI will be used for continuous variables. In this study, all statistical tests will be two-way, and a *P* value < 0.05 is considered valid.

## Discussion

In recent years, damage to the human body caused by PM2.5 has been a research topic of great interest, and many scientists have been looking for effective intervention methods. Currently, a standard PM2.5 protective mask is the most convenient method for individuals; however, wearing a PM2.5 protective mask for an extended period of time during work or physical activity often causes discomfort. People may wear PM2.5 masks incorrectly, and improper fit during activities may reduce their effectiveness [30].

PM2.5 exposure is one of several important factors that affect the differentiation status of CD4 + T helper cells [31, 32]. Studies have shown that the combined action of multiple cytokines leads to the polarization of TH1/TH2. IL-12 and IFN-γ stimulate naive CD4 + T cells to increase the expression of T-bet and STAT4 and to differentiate into TH1 cells. Similarly, IL-4 stimulates naive CD4 + T cells to increase the expression of STAT6 and GATA3 and to differentiate into TH2 cells [33]. TH1/TH2 polarization is involved in the progression of many diseases [33]. In this study, we will use the polarization state of TH1/TH2 as the main variable to examine the concentration of the primary cytokines in TH1 and TH2 immune cells to provide an objective explanation for the polarization of TH1/TH2 by omega-3 fatty acids. We will detect changes in ICAM-1 and IL-8 [34, 35], which have been identified as early indicators of atherosclerosis and vascular endothelial damage caused by PM2.5, to explain physiological mechanisms of PM2.5 damage to the vascular endothelium.

Omega-3 fatty acids, when used as nutritional supplements, have been shown to have beneficial effects on human health; however, research on the efficacy of omega-3 fatty acids on adverse health effects attributable to PM2.5 exposure has been concentrated on animal models. We will utilize employees of the Chengdu Metro system in China, who work mainly indoors, as research subjects for this study. Due to the rapid development of the Chengdu Metro in recent years, it is estimated that by the end of 2020, the total mileage of Chengdu Metro will rank fourth in China. Tens of thousands of subway workers have been exposed to PM2.5 for many years, and the disposable medical masks commonly worn by subway employees do not effectively protect employees from PM2.5 exposure. Few relevant data sets have been produced that examined changes in human biological markers associated with long-term exposure to PM2.5. Previous studies have focused on physiological damage caused by outdoor exposure to PM2.5, but in this experiment, we will examine PM2.5 exposure to employees inside the subway system. The relatively stable exposure environment of the enclosed subway system will improve the objectivity of the conclusions from this experiment.

### Trial registration

The trial was pre-registered at the China Clinical Trials Registry on September 9, 2020, under the registration number ChiCTR2000038065. See http://www.chictr.org.cn/index.aspx

## Trial status

Recruitment began on November 1, 2020, and the approximate date of completion is July 31, 2021. This protocol is based on protocol version 2.0 dated October 31, 2020.

## Modification of the protocol

Any changes to the study will be agreed by the project leader and the supervisor, and the project team members and the ethics committee will be notified before the changes can be made.

## Supplementary Information

**Additional file 1.** Chengdu Metro Line Map.

**Additional file 2.** SPIRIT 2013 Checklist.

**Additional file 3.** Informed Consent Form.

## Data Availability

Data sharing is not applicable to this article as no datasets are reported. The full protocol of this study will be provided by the corresponding author. The paper reporting the research results will disclose the availability of the data sets generated in the research. Upon a reasonable request, the author can access the complete agreement and model consent form.

## Abbreviations

CRF: Case report form
DSMB: Data and Safety Monitoring Board
DPA: Docosapentaenoic acid
DHA: Docosahexaenoic acid
EPA: Eicosapentaenoic acid
PM2.5: Fine particulate matter that has a diameter of less than 2.5 μm
PUFA: Polyunsaturated fatty acids
Res Man: Research Public Management platform
SPIRIT: Standard Protocol Items: Recommendations for Interventional Trials
SF-36: Short-Form Health Survey 36
