# Supplementary figures and images for: Effect of omega-3 fatty acids on TH1/TH2 polarization in individuals with high exposure to particulate matter ≤ 2.5 μm (PM2.5): a randomized, double-blind, placebo-controlled clinical study
Source: Trials. 2022 Feb 24;23:179. doi: 10.1186/s13063-022-06091-5 (PMC8867632; doi:10.1186/s13063-022-06091-5)

# Chengdu Metro Line Map

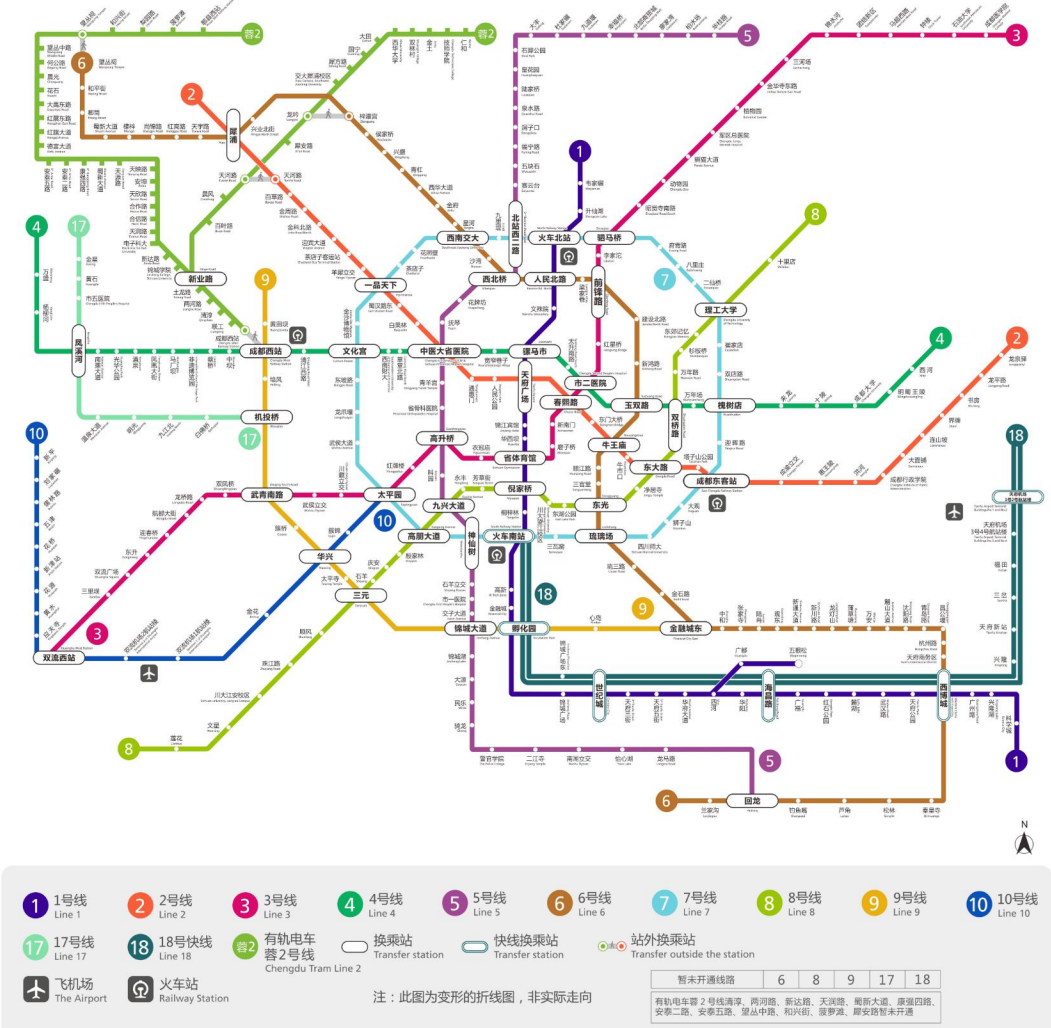

Supplement: Supplementary file 1 — Additional file 1. Chengdu Metro Line Map. [file 13063_2022_6091_MOESM1_ESM.pdf]
