# Supplementary material for: Effect of omega-3 fatty acids on TH1/TH2 polarization in individuals with high exposure to particulate matter ≤ 2.5 μm (PM2.5): a randomized, double-blind, placebo-controlled clinical study
Source: Trials. 2022 Feb 24;23:179. doi: 10.1186/s13063-022-06091-5 (PMC8867632; doi:10.1186/s13063-022-06091-5)
Supplement: Supplementary file 3 — Additional file 3. Informed Consent Form. [file 13063_2022_6091_MOESM3_ESM.docx]

**Informed Consent Form**

Name of participant: Gender: Age: Registry No:

Dear participant:

This study aims to discover the characteristics of changes in the TH1/TH2 polarization index in humans under high PM2.5 exposure, and to explore the clinical efficacy of Omega-3 polyunsaturated fatty acids intervention.

This is a clinical randomized controlled trial. If you agree to participate, you will be randomly assigned to placebo groups or intervention groups. The intervention group will get omega-3 fatty acids provided by Kirkland, Canada. The placebo group will get placebos made by placebo Experimental Center, School of Pharmacy, Chengdu University of TCM. Therapies in both groups are relatively safe. During the trial, if you have any unfavorable or unexpected signs, symptoms or diseases, you should report them to your doctor and they will take active measures.

During the course of the study, you will need to: (1) complete the entire 8 weeks of treatment, (2) during the study, you will fill out some questionnaires and answer some questions to evaluate the efficacy of the treatment regimen for 4 times in total (before treatment, after the final treatment, 12 weeks and 24 weeks after the final treatment), (3) collect your small amount of venous blood before and after treatment.

The participation in this study is entirely voluntary. Participants may be withdrawn at any time during the course of treatment without affecting the relationship between you and doctors. There is no loss in medical and economic aspects for you. You can also receive the effective treatment from doctors following routine medical procedures.

The research will strictly protect your privacy according to the principles of the Declaration of Helsinki. All information in this study will be kept confidential, and your private information will not appear in the research summary and published literature. This study has been ethically reviewed by the Chinese Ethics Committee of Registering Clinical Trials (Ethical review document number: ChiECRCT20190343).

**Voluntary Subject Statement**:

I have learned about the requirements of the clinical study in detail and the potential adverse reactions associated with it. I voluntarily participate in this clinical study, receive treatment on time, and fill out the relevant health questionnaire in accordance with the requirements of the research program. If adverse reactions occur, I will report to the doctor promptly. At the same time I know that the doctor will give positive response for my possible adverse reactions. I am also entitled to withdraw from the study at any time for any reason. However, if there are no special circumstances, I will cooperate with the doctor to complete the clinical study. My participation and the personal data in the trial are confidential. I agree with my doctor, the relevant regulatory authorities, and the ethics committee to review my information as required.

I (signature) relative (signature) (Relationship)

Date: D M Y

**Doctor's declaration**

I have fully explained the requirements of the clinical study in detail and the potential adverse reactions to the above participant/relative, and answered their questions. To the best of my knowledge, the participant/relative has been informed adequately and has consented.

Doctor’s signature: Date: D M Y

*In the event of inconsistency or discrepancy between the Chinese version and the English version, the Chinese language version shall prevail.*
